# Medical specialty certification exams studied according to the Ottawa Quality Criteria: a systematic review

**DOI:** 10.1186/s12909-023-04600-x

**Published:** 2023-08-30

**Authors:** Daniel Staudenmann, Noemi Waldner, Andrea Lörwald, Sören Huwendiek

**Affiliations:** University of Bern, Institute for Medical Education, Bern, Switzerland

**Keywords:** Medical education, Specialty certification examination, Validity, Reliability

## Abstract

**Background:**

Medical specialty certification exams are high-stakes summative assessments used to determine which doctors have the necessary skills, knowledge, and attitudes to treat patients independently. Such exams are crucial for patient safety, candidates’ career progression and accountability to the public, yet vary significantly among medical specialties and countries. It is therefore of paramount importance that the quality of specialty certification exams is studied in the scientific literature.

**Methods:**

In this systematic literature review we used the PICOS framework and searched for papers concerning medical specialty certification exams published in English between 2000 and 2020 in seven databases using a diverse set of search term variations. Papers were screened by two researchers independently and scored regarding their methodological quality and relevance to this review. Finally, they were categorized by country, medical specialty and the following seven Ottawa Criteria of good assessment: validity, reliability, equivalence, feasibility, acceptability, catalytic and educational effect.

**Results:**

After removal of duplicates, 2852 papers were screened for inclusion, of which 66 met all relevant criteria. Over 43 different exams and more than 28 different specialties from 18 jurisdictions were studied. Around 77% of all eligible papers were based in English-speaking countries, with 55% of publications centered on just the UK and USA. General Practice was the most frequently studied specialty among certification exams with the UK General Practice exam having been particularly broadly analyzed. Papers received an average of 4.2/6 points on the quality score. Eligible studies analyzed 2.1/7 Ottawa Criteria on average, with the most frequently studied criteria being reliability, validity, and acceptability.

**Conclusions:**

The present systematic review shows a growing number of studies analyzing medical specialty certification exams over time, encompassing a wider range of medical specialties, countries, and Ottawa Criteria. Due to their reliance on multiple assessment methods and data-points, aspects of programmatic assessment suggest a promising way forward in the development of medical specialty certification exams which fulfill all seven Ottawa Criteria. Further research is needed to confirm these results, particularly analyses of examinations held outside the Anglosphere as well as studies analyzing entire certification exams or comparing multiple examination methods.

**Supplementary Information:**

The online version contains supplementary material available at 10.1186/s12909-023-04600-x.

## Background

Patients rely on doctors for safe and effective medical care, yet preventable adverse events remain prevalent [1]. How can such events be avoided? One answer lies in professional assessments. Before being allowed to practice medicine independently, e.g., in their own private practice, doctors must pass a postgraduate exam which aims to test the skills, knowledge and attitudes relevant to their chosen medical specialty. Such medical specialty certification exams have long been used in many countries but differ greatly in their implementation. Historically, a simple oral examination by a senior colleague has often sufficed [2], but recent evidence supports the effectiveness of “triangulation”, a more multifaceted approach including assessment methods such as multiple choice questions (MCQs) and objective structured clinical examinations (OSCEs) [3].

The terminology of specialty exams differs substantially by country, even in the peer-reviewed literature published for a world-wide audience [4]. “Specialist medical assessment”, “board exam”, “postgraduate certification process”, “specialty certificate examination” and resulting acronyms are all commonly used. The institutions responsible for organizing the exams vary depending on country and medical specialty, as do the skills, knowledge and time spent training required as well as the privileges granted to a successful candidate [2].

In this article, we define a medical “specialty certification exam” as a high-stakes summative assessment of a candidate which takes place after completing postgraduate training such as residency, which is essential for career progression and – upon successful completion – typically allows the candidate to treat patients as an independent medical specialist. In the USA, 87% of physicians choose to get certified despite certification being voluntary [5]. Examples of specialty certification exams include the British “Royal College Membership exams”, the American “Board Certification” and the Swiss “Facharztprüfungen”.

Specialty certification exams are crucial to patient safety. Successful completion should guarantee the minimum level of competencies needed to diagnose and treat patients without a senior colleague readily available and formally responsible for ensuring the quality of the junior doctor’s treatment. Previous research shows that certified doctors generally provide better medical care than non-certified ones [6–9]. A systematic review by Lipner et al. shows that certification status is correlated with various clinical measures such as defibrillator complication rates or acute myocardial infarction mortality. In the majority of 29 studies, certified physicians provided better patient care [10]. To provide just one example, a study by Reid et al. shows certified physicians performing 3.3 percentage points higher on a quality performance composite than non-certified physicians across 23 specialties [9]. However, medical errors remain common overall [11] and cases of professional misconduct are regularly discussed in the media [12]. In one retrospective study, patients received only 54.9% of recommended basic care [13]. In American hospitals alone, medical errors are estimated to cause over 400′000 premature deaths per year [14], making it the third highest cause of death [15]. As the final examination of legally required formal education in many countries, specialty certification exams provide the last opportunity to identify physicians who do not (yet) qualify for unsupervised practice. They therefore play a crucial role in publicly guaranteeing practicing physicians’ competence.

In this study, we use the “Criteria for Good Assessment: Consensus Statement and Recommendations” from the Ottawa 2010 Conference (“Ottawa Quality Criteria”) to evaluate different medical specialty exams. This consensus statement was developed by a working group of medical assessment experts from various countries including Norcini et al. [16] and revised in 2018 [17]. They recommend the following seven criteria (Table 1):

**Table 1:** Framework for good assessment according to the consensus statement and recommendations from the Ottawa 2010 conference [16]

Criterion	Explanation
Validity or Coherence	The results of an assessment are appropriate for a particular purpose as demonstrated by a coherent body of evidence
Reproducibility, Reliability or Consistency	The results of the assessment would be the same if repeated under similar circumstances
Equivalence	The same assessment yields equivalent scores or decisions when administered across different institutions or cycles of testing
Feasibility	The assessment is practical, realistic, and sensible, given the circumstances and context
Educational Effect	The assessment motivates those who take it to prepare in a fashion that has educational benefit
Catalytic effect	The assessment provides results and feedback in a fashion that motivates all stakeholders to create, enhance, and support education; it drives future learning forward and improves overall program quality
Acceptability	Stakeholders find the assessment process and results to be credible

### Previous research

Given how important specialty certification exams are, there is a surprising lack of evidence pertaining to their efficacy [18]. Current literature often focuses on subspecialty specific exams in individual countries. To the best of our knowledge, the last systematic review was published in 2002 by Hutchinson et al. The authors searched different databases for studies published between 1985 and 2000, initially found 7705 and excluded all but 55 from their analysis. Hutchinson et al. then summarized each paper regarding any form of validity and reliability analyzed within. They remark on the paucity of published data, finding the under-representation of hospital specialties in particular “striking”. They call for a repeated analysis in the future and for increased openness “from many of the institutions that have a powerful and unopposed role in the career paths of doctors in training” [19].

Interest in the topic of effective medical education has increased sharply since then, yet there remains a gap in the literature concerning many specialties and countries. Hospital specialties are under-represented, while general or family practice predominates (covering 41 out of the 55 papers Hutchinson et al. identified). Hutchinson et al. found studies from only six countries, of which five were located in the Anglosphere [19]. Given their widespread use globally, the quality of most medical specialty exams remained to be scientifically studied according to either of the first two Ottawa Criteria (validity and reliability). To expand upon this research and address this gap in the literature, this systematic review focuses on collating up-to-date practices which have been analyzed according to any of the Ottawa Criteria from as many different countries and specialties as possible.

### Goal of this review

In this systematic literature review we aim to give an overview of the current evidence regarding specialty certification exams as studied according to any of the Ottawa Criteria of Good Assessment globally. We show which medical specialties, countries and examination formats have been analyzed regarding which of the Ottawa Criteria. This provides a point of reference for future researchers or medical specialty societies looking to study or further develop their exams.

The following research questions guide this systematic review:

1. Which medical specialty certification exams have been scientifically studied regarding the Ottawa Quality Criteria?
2. Which Ottawa Criteria were analyzed in these exams?
3. Which specialty certification exam has been studied most extensively in regard to the Ottawa Criteria?

## Methods and analysis

### Search strategy

Studies were compiled using the following seven databases: MEDLINE(R) ALL, EMBASE, APA PsycINFO and ERIC via Ovid, SCOPUS, the Cochrane Trial Library and Web of Science.

To reflect contemporary practice and continue from the timeframe used in Hutchinson et al.’s study, a search of the literature published between January 2000 and August 2020 was performed. The Population, Intervention, Comparison, Outcomes and Study (PICOS) design framework was used to establish the search strategy (see Table 2).

**Table 2:** PICOS framework

PICOS Elements	Characteristics
P—Population	Postgraduate medical trainees, physicians post completion of university studies
I—Intervention	High-stakes summative assessment of a medical specialist candidate which takes place after completion of postgraduate practical training (e.g. residency), which is necessary for career progression and typically allows the candidate to treat patients as an independent medical specialist upon successful completion Examples are the Swiss “Facharztprüfungen”, the British “Royal College Fellowship / Membership Exams” and the American “Board Certification”
C—Comparison	-
O—Outcome	Exam evaluation as measured by at least one of the Ottawa Criteria (validity, reproducibility, feasibility, equivalence, educational effect, catalytic effect, acceptability)
S—Study design	All study types

Because of the varying nomenclature of “specialty certification exams”, we expanded our search terms to cover over 20 variations and included the medical subject headings “Specialty Boards” and “Educational Measurement”. In addition, papers must reference the concept of medicine (e.g. “medic*”) and a form of evaluation criteria (e.g. “valid*”). Beyond this, we only include papers written in English and published between 2000 and 2020. The search terms described in Additional file 1 were used and adapted to the seven individual databases (see Additional file 1).

### Literature selection

The title, abstract and citation information of all results were retrieved from Ovid.com, Scopus.com, Webofknowledge.com and Cochranelibrary.com using the ris and Excel or csv format. They were imported into EndNote X9 and manually merged into an Excel file. The following information was made available separately to two researchers (DS and NW) for an initial round of screening: title, authors, year of publication and abstract. In this first round of screening all potentially valuable studies were included even if the fulfilment of certain criteria was questioned by one or both researchers. For instance, rather than examinations *of* physicians, many studies look at examinations *by* physicians. Others focus not on medical specialty exams but medical student exams, re-certification, maintenance of certification or formative workplace-based assessments. Papers describing assessments of other professions were similarly excluded (e.g. physician’s assistants, nurses and pharmacists). Manuscripts that weren’t published as complete scientific studies (e.g. conference papers, letters, editorials, reviews) were also excluded in this round, as were papers unavailable in English. In the second round the full text papers were assessed individually by DS and NW to determine whether the pre-selected papers fit the research questions. Here, papers were more likely to be excluded due to a lack of clarification of which examination method or methods were analyzed, because they assess exams which are administered almost immediately after candidates leave university or do not allow candidates to treat patients independently upon successful completion. Cases in which independent reviewers came to different conclusions were discussed bilaterally. If no agreement was found, a third reviewer (AL) was consulted for final judgement.

### Analysis

All results and interpretations pertaining to the Ottawa Quality Criteria were extracted from the included papers and categorized according to the seven criteria. DS and NW initially performed this step collaboratively to ensure they were able to reach consistent results, later extractions were then performed independently. Papers in which the relevant data or categorization was complex or unclear were discussed until agreed upon by DS, NW, and AL (see Additional file 2).

For further analysis, information about the country or countries studied, medical specialty or specialties, examination method or combination of methods as well as further relevant details about the examination were retrieved from the full text of all included papers. Where papers lacked such details, they were supplemented where possible by searching online. Details published e.g. by the medical society in question were added to the final overview, and the source of this additional information was included for reference. Papers were also shortly summarized for the convenience of the reader.

The methodological quality and relevance to this review’s research questions was evaluated for each study using the appraisal criteria adapted from the Medical Education Research Study Quality Instrument (MERSQI) and the “Criteria for the qualitative assessment of scientific publications” [20, 21]. The evaluation consists of the following six criteria: (1) Is the study design suited to answering the question studied? (2) Is the method described so that replication is possible without further information? (3) Is the interpretation coherent? (4) Does the study analyze at least 50 exam candidates? (5) Does it analyze more than one exam? (6) Does it analyze the entire exam(s)? Papers received one point if yes, zero if no or unclear.

Lastly, the data extracted was used to search for the specialty certification examination or examinations that were most extensively studied regarding the Ottawa Criteria by counting the number of separate Ottawa Criteria investigated as well as number of individual studies in cases where multiple papers were published that analyze the same exam.

## Results

**Fig. 1:**
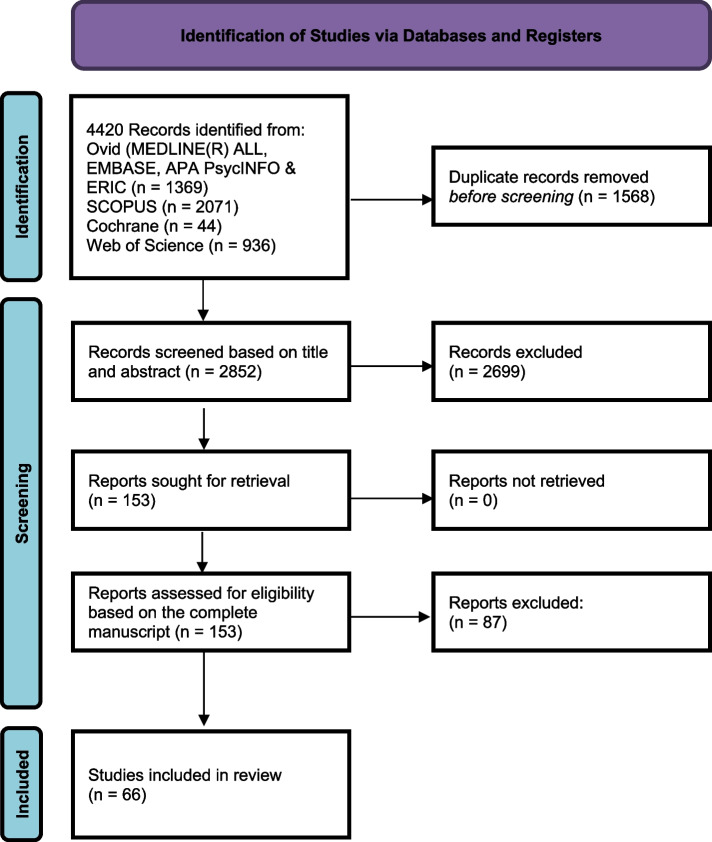
PRISMA flow diagram

The inclusion and exclusion process is visualized according to the PRISMA flow diagram (see Fig. 1). Out of 4420 hits, 66 studies were included for data analysis.

In the 66 papers, over 43 different exams and more than 28 specialties from 18 jurisdictions are assessed.

### Overview of studies

All included studies are sorted by medical specialty and country in Table 3. The Ottawa Criteria analyzed therein are marked ( +), those not analyzed (0). The right-most column shows how many of the six metrics studies fulfilled on the quality assessment tool. The examination formats used are listed, with the focus of the studies marked in bold. A complete overview including short summaries of all studies and the relevant findings can be found in the additional Excel spreadsheet (see Additional file 2).

**Table 3:** Overview of studies

Author, year	Medical specialty certification exam	Examination methods	Validity	Reproducibility	Equivalence	Feasibility	Educ. effect	Catal. effect	Acceptability	Quality metrics
**Berkenstadt et al., 2006 **[22]	Anesthesiology—Israel	Oral and **OSCE**	+	+	0	0	0	0	+	5
**Sun et al., 2019 **[23]	Anesthesiology—USA	MCQ, oral (**SOE** & OSCE)	+	+	0	0	0	0	0	4
**Warner et al., 2020 **[24]	Anesthesiology—USA	MCQ, oral (SOE & **OSCE**)	0	+	0	+	0	0	+	4
**Warner et al., 2020 **[25]	Anesthesiology—USA	MCQ, oral (SOE & **OSCE**)	0	0	0	+	0	0	0	4
**Gali et al., 2011 **[26]	Cardiology—Argentina	**MCQ (SBA)**, oral (with real patients)	+	0	0	0	0	0	+	3
**Tan et al., 2008 **[27]	Clinical Oncology—United Kingdom	**MCQ (SBA)**, oral (structured & clinical)	+	0	0	0	0	0	0	5
**O'Leary, 2015 **[28]	Emergency Medicine—Australia, New Zealand	Written & **Clinical (OSCE**)	+	+	0	0	0	0	0	3
**Bianchi et al., 2003 **[29]	Emergency Medicine—USA	MCQ, **oral (simulated cases)**	0	+	0	0	0	0	0	4
**Slovut et al., 2015 **[30]	Endovascular Medicine—USA	**MCQ**	+	+	0	0	0	0	+	4
**Khafagy et al., 2016 **[31]	Family Medicine—Egypt	**MCQ, SBA**, clinical assessment	+	+	0	0	0	0	0	5
**Weingarten, et al., 2000 **[32]	Family Medicine—Israel	MCQ, **oral** (structured)	0	+	0	0	0	0	0	5
**O'Neill et al., 2011 **[33]	Family Medicine—USA	**Written (MCQ, SBA)**, oral (case discussion)	+	0	0	0	0	0	0	4
**O'Neill et al., 2019 **[34]	Family Medicine—USA	**Written, oral (case discussion)**	+	0	0	0	0	0	0	4
**Greco et al., 2002 **[35]	General Practice—Australia	Applied Knowledge Test (MCQ), Key Feature Problem (KFP), **Objective Structured Clinical Examination (OSCE)**	+	0	0	0	0	0	0	4
**Munro et al., 2005 **[36]	General Practice—United Kingdom	**Written (free text answers** and MCQ), Oral, consultation skills	+	+	0	0	0	0	0	5
**Simpson et al., 2005 **[37]	General Practice—United Kingdom	Written (free text answers and MCQ), **Oral**, consultation skills	+	0	0	0	0	0	0	4
**Sandars et al., 2004 **[38]	General Practice—United Kingdom	Written **(free text answers** and MCQ), Oral, consultation skills	+	+	+	0	0	0	0	2
**Dixon, 2005 **[39]	General Practice—United Kingdom	Written (free text answers and **MCQ**), Oral, consultation skills	+	0	0	0	+	+	+	3
**Siriwardena et al., 2006 **[40]	General Practice—United Kingdom	Written (free text answers and MCQ), Oral, **consultation skills**	+	0	0	0	0	0	0	4
**Dixon, 2003 **[41]	General Practice—United Kingdom	**Written (free text answers and MCQ), Oral, consultation skills**	0	0	0	0	+	+	+	4
**Wass et al., 2003 **[42]	General Practice—United Kingdom	Written (free text answers and MCQ), **Oral (structured)**, consultation skills	+	+	0	0	0	0	0	4
**Dixon et al., 2015 **[43]	General Practice—United Kingdom	**Applied Knowledge Test (MCQ);** Clinical Skills Assessment (OSCE); Workplace Based Assessment (WBA)	+	0	0	0	+	0	+	4
**Partridge, 2008 **[44]	General Practice—United Kingdom	**Written (free text answers and MCQ)**, Oral (structured), consultation skills	+	0	0	0	0	0	+	5
**Dixon et al., 2007 **[45]	General Practice—United Kingdom	Written (free text answers and **MCQ**), Oral (structured), consultation skills	+	+	0	0	0	0	+	3
**Bourque et al., 2020 **[46]	Internal Medicine—Canada	**MCQ,** OSCE	0	+	0	0	0	0	0	4
**Chierakul et al., 2010 **[47]	Internal Medicine—Thailand	Written and **clinical** (real patients)	+	+	0	0	0	0	0	4
**McManus et al., 2003 **[48]	Internal Medicine—United Kingdom	**Part 1: MCQ (true–false)**, Part 2: unclear	0	+	0	0	0	0	0	4
**McManus et al., 2013 **[49]	Internal Medicine—United Kingdom	Part 1: MCQ (SAQ); Part 2: Written**; Part 2 Clinical (PACES)**	+	0	0	0	0	0	0	5
**McManus et al., 2006 **[50]	Internal Medicine—United Kingdom	Part 1: MCQ (SAQ); Part 2: Written**; Part 2 Clinical (PACES)**	0	+	0	0	0	0	0	5
**Atsawarungruangkit, 2015 **[51]	Internal Medicine—USA	**MCQ (SBA)**	+	0	+	0	0	0	0	5
**Marques et al., 2018 **[52]	Multiple—Portugal	**Curriculum analysis, practical (real patient exam, discussion) and theoretical tests (Oral or MCQ)**	+	+	0	0	0	0	0	6
**Burch et al., 2009 **[53]	Multiple—South Africa	**Short-answer question test (SAQT), Data interpretation test (DIT), real patient encounters (PE)**	+	0	0	0	0	0	0	4
**Burch et al., 2008 **[54]	Multiple—South Africa	Written: **MCQ, short-answer question tests (SAQT)** Oral: **Real patient encounters followed by oral test**, Data interpretation Test (DIT), PE (real patient encounters and oral exam)	0	+	0	0	0	0	0	6
**Cookson, 2010 **[55]	Multiple—United Kingdom	**MCQ (SBA)**	+	+	0	+	+	0	+	4
**Mucklow, 2011 **[56]	Multiple—United Kingdom	**MCQ (SBA)**	+	+	0	0	0	0	+	6
**Raddatz, et al., 2012 **[57]	Not specified—USA	Not specified	+	0	0	0	0	0	0	3
**Lunz et al., 2008 **[58]	Not specified—USA	**Oral (real or realistic patient cases)**	0	+	0	0	0	0	0	5
**Houston et al., 2009 **[59]	Not specified—USA	**Oral (structured)**	+	+	0	0	0	0	0	3
**Mathysen et al., 2013 **[60]	Ophthalmology—Europe	written: **MCQ (true–false)**, oral (structured)	+	+	0	0	0	0	0	5
**Mathysen et al., 2013 **[61]	Ophthalmology—Europe	written: **MCQ (true–false)**, oral (structured)	+	+	0	+	0	0	0	5
**Chow et al., 2017 **[62]	Palliative Medicine—China, Hong Kong	Dissertation Appraisal Examination, **Oral Examination**	+	+	0	0	0	+	0	3
**Althouse et al., 2009 **[63]	Pediatrics—USA	**MCQ (SBA)**	+	0	0	0	0	0	0	5
**Emadzadeh et al., 2017 **[64]	Pediatrics & Gynecology—Iran	MCQ, **OSCE**	0	0	0	0	0	0	+	4
**Raddatz et al., 2017 **[65]	Physical Medicine and Rehabilitation—USA	**MCQ**, oral (structured)	+	+	0	0	0	0	0	4
**Tibbo et al., 2004 **[66]	Psychiatry—Canada	MCQ,** oral/clinical (real patients)**	0	0	0	+	0	0	+	4
**Tong et al., 2018 **[67]	Radiology—Europe	**Written** (SBA, MAQ, Order), **Oral** (unclear)	+	0	0	0	+	0	+	5
**Yeung et al., 2013 **[68]	Radiology—United Kingdom	MCQ, rapid reporting session, long-cases reporting session,** oral (structured)**	0	+	0	+	0	0	0	4
**Yeung et al., 2011 **[69]	Radiology—United Kingdom	MCQ, SBA, **reporting session, rapid reporting session, oral (structured)**	+	+	0	0	+	0	+	4
**Yang et al., 2013 **[70]	Radiology—USA	Written (unclear), **oral (structured)**	0	+	0	0	0	0	0	4
**Kerridge et al., 2016 **[71]	Radiology—USA	**MCQ,** oral (structured)	+	0	0	+	+	0	+	1
**Yang et al., 2010 **[72]	Radiology—USA	**MCQ**, oral	+	0	+	0	0	0	0	4
**Pascual-Ramos et al., 2018 **[73]	Rheumatology—Mexico	MCQ, **OSCE**	+	0	0	+	0	0	0	4
**Smith et al., 2007 **[74]	Rural and Remote Medicine—Australia	**Written (MCQ, SBA), StAMPS,**	+	+	0	+	+	0	+	4
**Beasley et al., 2013 **[75]	Surgery (9 different surgical specialties)—Australia, New Zealand	**Written and clinical/viva**	+	+	0	0	0	0	0	6
**De Montbrun, 2016 **[76]	Surgery (colon and rectal)—USA	MCQ, oral, **COSATS (technical skill tasks)**	+	+	0	+	0	0	0	4
**Lineberry et al., 2020 **[77]	Surgery (endoscopic)—USA	MCQ, **manual skills test**	+	+	0	+	0	0	0	4
**Motoyama, et al., 2020 **[78]	Surgery (esophageal)—Japan	**Clinical experience**	+	0	0	0	0	0	0	5
**De Montbrun, 2017 **[79]	Surgery (general and colorectal)—USA, Canada	**Multiple**	+	+	0	+	0	0	0	5
**Crisostomo, 2011 **[80]	Surgery (general)—Philippines	MCQ,** oral (structured)**	+	+	0	0	0	0	0	4
**Rhodes et al., 2007 **[81]	Surgery (general)—USA	**MCQ, oral**	+	0	0	0	+	0	0	3
**Cundy, 2012 **[82]	Surgery (orthopaedic)—Australia	Written and **clinical/viva** (real patients)	0	+	0	0	0	0	+	1
**Hohmann et al., 2018 **[83]	Surgery (orthopaedic)—Australia, UK, South Africa & Canada	**Multiple: Written (MCQ, essays, short questions), oral (viva voce), operative sessions, clinical sessions or OSCEs**	+	0	+	0	0	0	0	5
**Gillis et al., 2020 **[84]	Surgery (orthopaedic)—Canada	**S-OSCE**	+	0	0	+	0	+	0	3
**Ullmann et al., 2006 **[85]	Surgery (plastic)—Israel	MCQ, **oral** (unstructured)	+	0	0	0	0	0	+	4
**Dwyer et al., 2020 **[86]	Surgery (sports medicine)—Canada	MCQ, **OSCE**, in-training evaluation, surgical logbook, **intraoperative and cadaveric assessment**	+	+	0	0	+	0	0	4
**Payne et al., 2011 **[87]	Urology—United Kingdom, Ireland	**MCQ (SBA, EMI), oral (structured)**	+	0	0	0	0	0	+	6

### Location

**Fig. 2:**
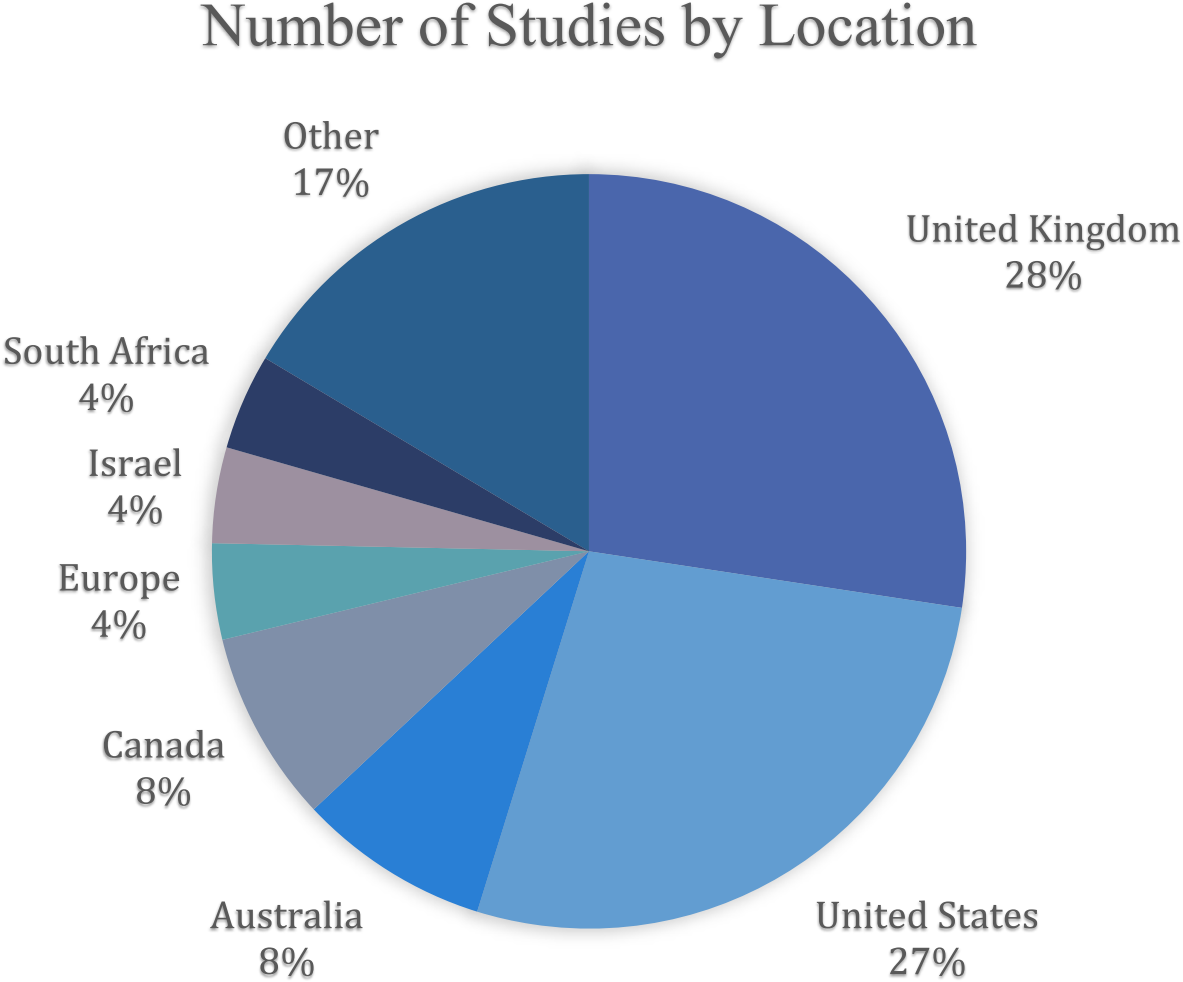
Number of studies by location

A large majority of our search results examine the specialty certification exams used in English-speaking countries, with 77% of papers focusing on the UK, USA, Australia, Ireland, Canada, or South Africa. By far the two most frequently studied countries are the United Kingdom and the United States, together comprising 55% of eligible papers (20 publications each, see Fig. 2). Other locations studied include Israel, China Hong Kong, Argentina, Egypt, Iran, Japan, Mexico, the Philippines, Portugal, and Thailand. A minority of exams are not specific to only one country: three papers look at European exams, two at Australia and New Zealand, one at the USA and Canada, and one at the UK and Ireland. A single study compares the exams across multiple countries [88].

### Specialty

**Fig. 3:**
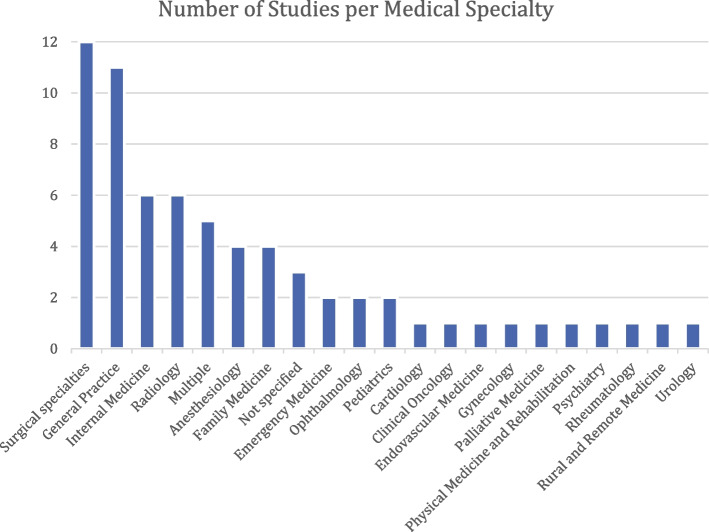
Number of studies by medical specialty

The certification exams used in the specialty of General Practice are the most frequently studied, with 11 studies focusing on this domain. Various kinds of surgical specialties are studied in 12 different publications. Internal Medicine is analyzed in six different studies. A further six studies assess the exams used in Radiology. Anesthesiology and Family Medicine are the medical specialties under consideration in four studies each. Emergency Medicine, Ophthalmology, and Pediatrics are each analyzed in two studies. One study has been published about each of the following medical specialty certification exams: Cardiology, Clinical Oncology, Endovascular Medicine, Gynecology, Palliative Medicine, Physical Medicine and Rehabilitation, Psychiatry, Rheumatology, Rural and Remote Medicine, and Urology (see Fig. 3). Five studies evaluate multiple medical specialties. Three studies fail to specify which specialty exam is under analysis.

### Methodological quality and relevance assessment

**Fig. 4:**
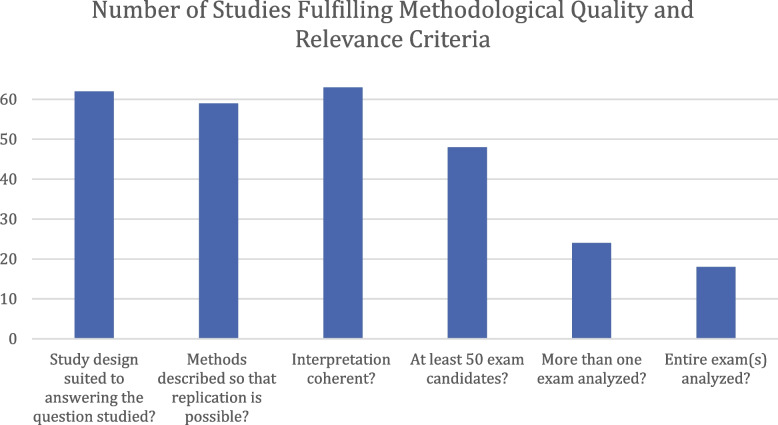
Number of studies fulfilling methodological quality and relevance criteria

Eligible papers receive an average of 4.15 out of the six possible points for relevance and methodological quality. 94% (62/66, see Fig. 4) of studies receive a point for criterion 1 (“Is the study design suited to answering the question studied?”), 89% (59/66) for criterion 2 (“Is the method described so that replication is possible without further information?”), and 95% (63/66) for criterion 3 (“Is the interpretation coherent?”). The number of candidates analyzed is at least 50 in 73% (48/66) of studies (criterion 4). 36% (24/66) of studies compare multiple exams (criterion 5). Finally, 28% (18/66) of the included studies analyze the entire exam(s) (criterion 6). Many focus on only a subset of the specialty certification exam, though some studies also receive zero points on this metric since it is unclear what the entire specialty certification exam under consideration consists of.

### Examination methods

The nomenclature varies widely across different examination modalities. The most common methods used include multiple choice questions (MCQ), structured oral exams with expert discussions and objective structured clinical examinations (OSCE). Essay questions, dissertation appraisal or clinical experience are less frequently evaluated in the studies. Few medical specialty certification exams only use a single examination method. A large majority of studies published therefore focus on exams using a combination of different modalities, comprising of at least one written and one oral method.

### Ottawa criteria

**Fig. 5:**
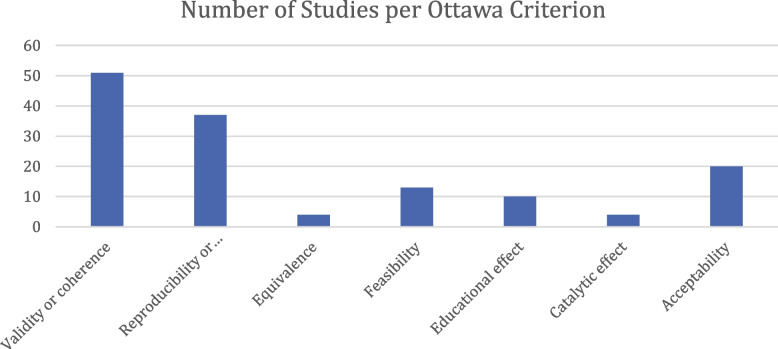
Number of Studies per Ottawa Criterion

On average, studies examine 2.1 of the 7 Ottawa Criteria. The most frequently studied criterion is validity (51/66 studies), followed by reliability (37/66) and acceptability (20/66). Feasibility is a topic of analysis in 13 papers. Equivalence and catalytic effect are least commonly researched, with 4 studies mentioning results belonging to those categories each (see Fig. 5).

**Fig. 6:**
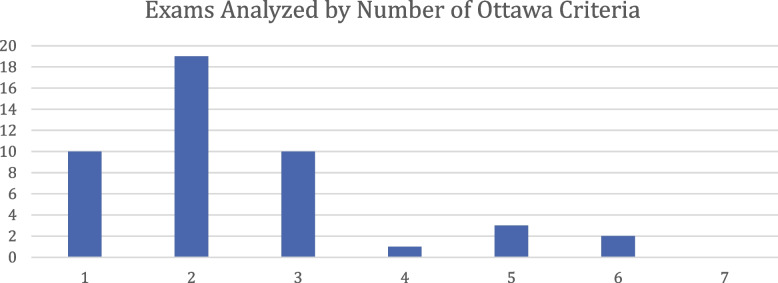
Exams Analyzed by Number of Ottawa Criteria

No medical specialty certification exam has been analyzed in respect to all seven Ottawa Criteria. Even when collating evidence from multiple studies, only 16 out of 46 exams have been analyzed in respect to three or more. Three exams have been analyzed in respect to five, and two exams in respect to six of the seven Ottawa Criteria (see Fig. 6).

### The most extensively studied exam – The MRCGP

The Membership of the Royal College of General Practitioners (MRCGP) exam is the most extensively studied specialty certification exam regarding the Ottawa Criteria, with 10 different papers published between 2000 and 2020. Apart from the feasibility criterion, all Ottawa Criteria are covered by the literature.

The MRCGP examination aims to test the skill and knowledge of a doctor who “has satisfactorily completed specialty training for general practice and is competent to enter independent practice in the UK without further supervision” [89]. Although changes continue to be made to the exam’s format, in nearly all the included studies it is described as consisting of the following four parts: a written exam (called “written paper”) made up of free text answers, a multiple-choice question exam, an oral exam and a video section examining consultation skills [37–39, 41, 42]. The individual research studies published about the MRCGP are presented in more detail below.

Dixon [41] surveyed registrars about their views on the various MRCGP modules and their effects on learning. He found that candidates perceived study groups of fellow registrars as particularly helpful to prepare for the written and oral components, and feedback from trainers as especially useful for the consultation skills video component. Many said they had read more review articles but not original articles as preparation. Most candidates believed that preparing for the oral module increased their understanding of moral and ethical principles.

The “written paper” in the MRCGP examination aims to test candidates’ problem-solving skills, knowledge of current literature and critical appraisal skills of study methodology. As described by Sandars et al. [38], candidates are awarded three and a half hours to read three literature extracts and write concise “notes” answering 12 questions, usually about the studies’ methodology and how this relates to a given scenario relevant to general practitioners (GPs). Munro et al. [36] show these examiner-marked “Free Text Answers” achieve relatively high measures of reliability with Cronbach’s alpha consistently lying between 0.85 and 0.88. Dixon [39] asked candidates to rate their impression of the question formats, finding that the single best answer (SBA) format was rated easiest, and the treatment algorithm completion, extended matching and summary completion questions as more difficult. Summary completion questions were also criticized for testing language ability. Overall, the acceptability of the written paper was high among candidates, although a majority believe this module also contained inappropriate questions. The written paper module of the MRCGP exam seemed particularly helpful in encouraging candidates to regularly read journal articles. Partridge [44] supports this view and further emphasizes the importance of critical appraisal skills for the written paper. A large majority of the candidates were generally satisfied with this part of the exam and found the questions to be clear and relevant to General Practice.

The “Multiple Choice Paper” (MCP) uses a number of question formats including SBA, images and extended matching questions to test the breadth and depth of candidates’ knowledge. Dixon et al. [45] asked GP trainers to sit a shortened version of the MCP, finding that they significantly outperform registrars even with no preparation on overall scores as well as questions specifically related to General Practice and practice administration. Trainers did not manage to answer questions related to research methodology or critical appraisal significantly more often than candidates. Accordingly, despite other question topics being perceived as easy, research methodology and critical appraisal questions were rated as difficult by most trainers. Dixon et al. [43] also summarize candidates’ views on this part of the exam, finding that it was perceived to succeed in its aim of being a fair test of candidates’ knowledge and relevant to General Practice. The topics research and statistics were found to be most difficult by those candidates. However, they did not achieve lower mean scores in these fields. Small adjustments such as adding a calculator and allowing ten minutes extra time were made in response to this feedback.

The “Oral Exam” aims to test candidates’ decision-making skills and professional values using two 20-min oral exams with a pair of examiners and five topics each. The examiners chose their own questions. Wass et al. [42] find the reliability coefficients to be lower than required (intercase 0.65, pass/fail 0.85) and recommend increasing the testing time and number of topics covered, suggesting five oral exams with one examiner each. This would increase intercase reliability to 0.78 and pass/fail reliability to 0.92. Simpson et al. [37] also looked at the oral exam, arguing that “the assessment of professional values was largely examined at the level of knowledge and comprehension, with few examiners encouraging candidates to justify their expressed viewpoint or allowing them to demonstrate how they might use these values to support their decision making.”

For the “Consulting Skills Assessment”, candidates are asked to submit videos of themselves interacting with seven real patients. They can choose those seven consultations to best demonstrate 15 performance criteria and are then rated by seven independent GPs trained for this assessment. Siriwardena et al. [40] compared this module with the ‘observing patient involvement’ (OPTION) scale – an independently validated scale for shared decision making – finding that it predicts both the performance criterion ‘sharing of management options’ as well as overall MRCGP results.

## Discussion

This systematic review tackles an important question in current medical education research: How can we credibly test and certify physicians’ competence? Specialty certification exams are crucial for patient safety, candidates’ career progression and accountability to the public, yet evidence to their quality has thus far been lacking. By searching seven different databases and using a wide variety of possible variations in search terms, we collate a comprehensive outline of the research regarding studied Ottawa Quality Criteria in specialty certification exams published in the past twenty years. 66 studies were included. Reliability, validity, and acceptability are the criteria most frequently studied in respect to specialist exams in this literature. As was the case in the previous literature review by Hutchinson et al., the largest body of evidence is centered on the UK and USA as well as the General Practice specialty [19]. However, we document a large increase in the number of different countries, medical specialties and Ottawa Criteria studied during the past twenty years.

The exact nomenclature used to describe examination quality indicators in the literature and the relative emphasis of the authors may vary, yet there exists widespread agreement as to which qualities good examinations must fulfill. Medical specialty certification especially must be valid, reliable, and objective. When repeated, they ought to give similar results and therefore be reproducible, independently of factors such as examiner bias [3]. Furthermore, such assessments must be feasible, remain as cost-efficient as possible, and provide adequate feedback *for* and *of* learning [90]. All these aspects are covered through the seven criteria for good assessment from the Ottawa Conference chosen for this review: validity, reliability, equivalence, feasibility, acceptability, catalytic and educational effect. Due to the high-stakes summative nature of specialty certification exams, the focus often lies on ensuring validity and reliability rather than educational or catalytic effect. This trend is reflected in the number of studies found analyzing each criterion.

We can see how Ottawa Criteria sometimes conflict with each other. For instance, although acceptability among candidates may suffer if an examination program neglects the provision of constructive feedback, the priority for an institution organizing the exam may lie on credibly signaling to the public, healthcare institutions and patients that a passing candidate is ready for independent practice. How such tradeoffs among quality criteria may be improved with limited resources can be studied with the feasibility criterion. Feasibility, including financial cost associated with different examination methods, is thus a major concern regarding high-quality specialty certification exams organized in resource-constrained contexts. Despite its relevance, we observe a relative scarcity of studies concerning this criterion [24, 25, 55, 61, 66, 68, 71, 73, 74, 76, 77, 79, 84, 91].

According to Miller’s framework for assessing clinical skills, competence and performance, clinical assessment can be conceptualized in four progressive levels: the learner proceeds through “knows,” “knows how” and “shows how” to “does” [92]. It is the first that is easiest to test reliably on a written exam, yet proficiency at the highest level must be reached before a candidate can be certified for independent practice. Unless they can demonstrate their knowledge, skills, as well as attitudes, we cannot be sure this is the case: “No single assessment method can provide all the data required for judgment of anything so complex as the delivery of professional services by a successful physician” [92]. All examination methods face limitations on at least one Ottawa Quality Criterion and cannot be expected to cover all levels of Miller’s framework [93]. Well-designed specialty certification exams manage to also check the higher levels of Miller’s pyramid, and thereby make the exam conditions match the reality of working as a certified physician more closely [3, 92, 94].

The necessity of combining different assessment methods in specialty certification exams was highlighted for US internal medicine residents specifically in a 1998 non-systematic review article by Holmboe et al. [95]. They summarize studies published between 1966 and 1998 and argue that since the written American Board of Internal Medicine (ABIM) certification exam alone is insufficient to adequately assess clinical competence, it should be supplemented by other examination methods in the clinic such as rating scales of interpersonal skills and attitudes, medical record audits, clinical evaluation exercises (CEX) and standardized patient exams. More recent work has further recommended expanding assessment to include competencies such as teamwork and population care [96, 97]. It is therefore encouraging that a majority of medical specialty certification exams analyzed in this review use triangulation methods and e.g. complement multiple choice questions (“knows” and “knows how”) with OSCEs (“shows how”) and workplace based assessments (“does”) [22–29, 31, 35–46].

Single exams only at the end of an educational period can lead candidates to ignore the feedback given [93]. Further development of the medical specialty certification process may therefore consist of additional longitudinally administered assessments (e.g. workplace-based assessments).

This approach has seen increasing support in so-called programmatic assessments of competency-based medical education (CBME). In this system-based approach to assessment design, pass-fail decisions are based on a portfolio containing datapoints created by multiple assessors and assessments [90, 98]. The time of examination gets decoupled from the time of a high-stake decision such as promotion or graduation [99]. The aim is for candidates to gain valuable information from both a mentor’s critical feedback, support, and self-reflection without such programs becoming overly bureaucratic or time-consuming [100, 101]. A combination of longitudinally repeated workplace-based assessments and structured examinations as summarized in this article seems most promising in supporting this goal as well as providing crucial data points for the high-stakes decision on qualification for unsupervised practice.

Since most papers analyzed in this review focus exclusively on one aspect of the exam, it is often not possible to comprehensively evaluate the entire specialty certification exam. Few studies look at multiple examination formats and compare them [24, 27, 35, 41, 54, 69, 86–88]. Strengths and weaknesses identified with just one assessment method may therefore be compensated for in another part of the exam without this effect being accounted for in the literature.

It is possible that, due to the time span under consideration, examination formats have changed in the time since the included studies have been conducted, and certain critiques expressed in a paper may have already been incorporated into practice. This is the case with the Membership of the Royal College of General Practitioners (MRCGP UK) exam, which was used to illustrate the literature on a particularly well researched medical specialty certification exam. At the time of study, this consisted of a written exam made up of free text answers, a multiple-choice question exam, an oral exam and a video section examining consultation skills.

The RCGP has since decided to change the formats to further improve the examination. Due to potential problems pertaining to validity and reliability – particularly inter-rater reliability – the use of oral examinations has been discontinued in many countries including the UK in favor of more clearly structured examination formats. The written exam has been complemented with an OSCE-based Clinical Skills Assessment (CSA) and a Workplace Based Assessment (WPBA). In the CSA, patients are played by trained and calibrated actors which allows for the simulation of real-life consultations [102]. The goal of the WPBA is to evaluate candidates in their day-to-day practice and provide constructive feedback as well as specifically assess aspects of professional behavior that are difficult to measure using only the written exam and the CSA [103]. Further adaptations were introduced on a temporary basis due to the Covid pandemic [104]. Exactly how the current version of the MRCGP exam compares to the previous examination format on various assessment criteria has not been shown in the existing literature.

Although the proven validity and reliability of OSCE-style examination formats has increased their attractiveness to institutions around the world, a possible downside may relate to their acceptability. “Examiners generally do not like structured assessments” due to the lack of spontaneity and flexibility to adapt the assessment to the abilities of the candidate [69]. Nevertheless, the current combination of examination formats arguably allows for a comparatively comprehensive candidate assessment: written exams test knowledge, can be highly standardized and are easily feasible, OSCEs fulfill standardization requirements while allowing for an assessment of the “shows how” level of test learning, and WPBAs complement these methods by providing more realistic, personalized assessment data.

### Strengths & limitations

With search terms covering a variety of possible synonyms of medical specialty certification exams, this review provides the most extensive and up-to-date overview thus far, allowing for an accurate picture of current medical specialty certification exams that have been scientifically evaluated in regard to any of the Ottawa Criteria globally. Together, these seven criteria cover vital aspects of assessment quality.

However, we find many exams have yet to be scientifically analyzed according to any of the Ottawa Quality Criteria. This means some countries and medical specialties are not included in this review. We find it is common for specialty certification exams or different examination formats to be scientifically studied across only a select few criteria or only pertaining to part of the examination. An overall quality ranking leading to a clear recommendation regarding which exam best achieves all seven Ottawa Criteria of Good Assessment could not be supported by the current literature regarding specialty certification exams.

Another limitation of this review is that literature published in languages other than English or exclusively in databases not included in our search is not included in this review. This disadvantages countries where the primary language is not English and may partially explain the predominance of literature about exams based in the UK, USA and other anglophone countries in our findings.

### Implications for practice

This systematic literature review provides an overview of medical specialty certification exams, the respective examination methods used, and their evaluation in respect to the Ottawa Criteria. It can thus assist those looking to improve the current specialty certification exams by showcasing the strengths and weaknesses of existing exams. Based on the findings of the papers presented in this systematic review, we can build upon the research most relevant to our medical specialty and learn from the strengths and weaknesses highlighted in examination formats studied in other countries. Certifying bodies looking to expand their current set of examination methods can find tried and tested methods in the research presented here. By collating the published research, this review can also guide readers deciding which specialty certification exams to accept in their jurisdiction. Finally, it offers an index of the leading researchers in this area, serving those looking to further collaborate or study a specific certification exam in respect to the seven Ottawa Criteria.

### Implications for future research

Further research should summarize how well exams fulfill all Ottawa Criteria and compare them accordingly. What is the best examination method to use in resource-constrained settings? Which medical specialty manages to test its candidates most reliably? And overall, regarding all seven Ottawa Criteria, what’s the best way to organize a medical certification exam? These kinds of research inquiries seem promising as they reflect the literature gaps highlighted in this review. Numerous studies comparing certified to non-certified doctors exist [105–107], yet studies linking the examination formats to the subsequent performance of certified compared to non-certified physicians would be more useful in deciding how best to structure a specialty certification exam. For instance, natural experiments when certifying bodies update their practices or cohort studies following doctors certified using different examination methods could look at varying outcomes in patient safety. Case reports highlighting the use of innovative new examination formats may also offer potential improvements to the established techniques. Further research should fill the gaps highlighted in this review regarding the examinations, countries and the Ottawa Criteria not yet studied, to allow for a holistic comparison across examinations.

Further research should use the seven Ottawa Criteria to focus on medical specialty certification exams in more non-English-speaking countries and a wider variety of specialties. They should make use of additional sources such as grey literature internal to certifying institutions and expert interviews to shed insight into less frequently studied Criteria such as feasibility. Establishing a common nomenclature which covers the pre-requisites, assessment methods and consequences of medical specialty certification exams would make future comparisons more straight-forward. Although the general quality of the studies we found was good, most of the current research analyzed only a fraction of the entire exam and did not compare different examinations. These approaches should be pursued to allow for a more comprehensive evaluation and better guide recommendations for future practice.

Overall, despite the increased interest over the past few decades outlined above, there continues to be an urgent need for more publicly available research to return the trust which the public places in the certification process of medical doctors.

## Conclusion

The past twenty years have seen a growing interest in the topic of patient safety and effective medical specialty certification exams. This is reflected in a growing number of studies analyzing medical specialty certification exams covering a larger variety of medical specialties, countries, and Ottawa Criteria. Medical specialty certification exams vary significantly between countries and are constantly adapted to changing circumstances through new examination formats. Due to their implications for patient safety, rising public scrutiny over medical self-regulation and their impact on candidate’s career opportunities, it is of paramount importance they be supported by a large body of evidence which demonstrates fulfillment of all seven Ottawa Criteria of good assessment. Due to their reliance on multiple assessment methods and data-points, aspects of programmatic assessment suggest a promising way forward in the development of effective medical specialty certification exams. To confirm and expand on these results, future research should focus on examinations held outside the Anglosphere, analyses of entire certification exams, and comparisons across examination methods.

### Supplementary Information

**Additional file 1.** Search terms.

**Additional file 2.** Overview of studies

## Data Availability

The dataset supporting the conclusions of this article is included within the article (and its additional files).

## Abbreviations

ABIM: American Board of Internal Medicine
CBME: Competency-Based Medical Education
CEX: Clinical Evaluation Exercise
CSA: Clinical Skills Assessment
GP: General Practitioner
MCP: Multiple Choice Paper
MCQ: Multiple Choice Question
MERSQI: Medical Education Research Study Quality Instrument
MRCGP exam: Membership of the Royal College of General Practitioners exam
OPTION scale: ‘Observing patient involvement’ scale
OSCE: Objective Structured Clinical Examination
PICOS: Population, Intervention, Comparison, Outcomes and Study
RCGP: Royal College of General Practitioners
SBA: Single Best Answer
WPBA: Workplace Based Assessment
